# Supplementary material for: Inorganic Polyphosphate Promotes Colorectal Cancer Growth via TRPM8 Receptor Signaling Pathway
Source: Cancers (Basel). 2024 Sep 28;16(19):3326. doi: 10.3390/cancers16193326 (PMC11476407; doi:10.3390/cancers16193326)
Supplement: Supplementary file 1 [file cancers-16-03326-s001.zip › Supplementary files prima parte proliferazione.pdf.pdf]

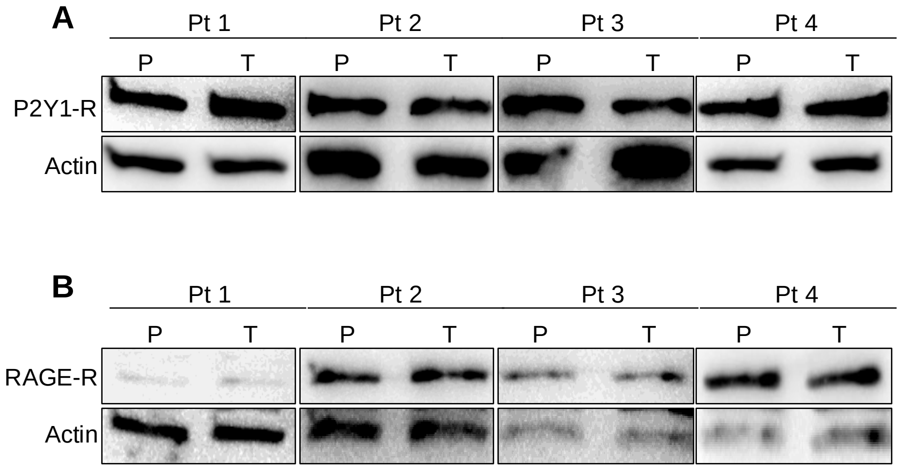
A
Pt 1
Pt 2
Pt 3
Pt 4
P
T
P2Y1-R
Actin
B
Pt 1
Pt 2
Pt 3
Pt 4
P
T
RAGE-R
Actin

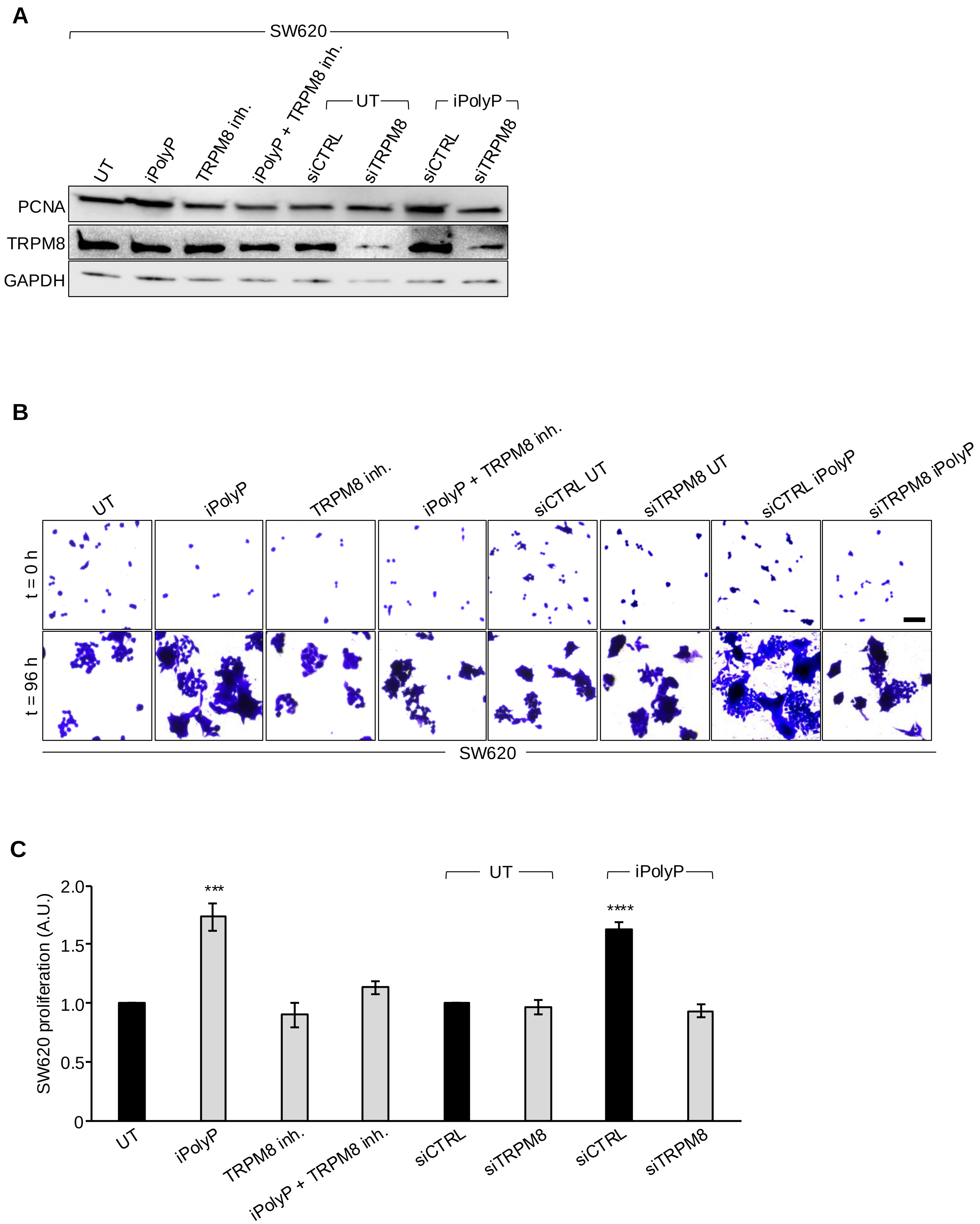
A
SW620
UT
iPolyP
TRPM8 inh.
iPolyP + TRPM8 inh.
UT
iPolyP
siCTRL
siTRPM8
siCTRL
siTRPM8
PCNA
TRPM8
GAPDH
B
UT
iPolyP
TRPM8 inh.
iPolyP + TRPM8 inh.
siCTRL UT
siTRPM8 UT
siCTRL iPolyP
siTRPM8 iPolyP
t = 0 h
t = 96 h
SW620
C
SW620 proliferation (A.U.)
2.0
1.5
1.0
0.5
0
***
****
UT
iPolyP
UT
iPolyP
TRPM8 inh.
iPolyP + TRPM8 inh.
siCTRL
siTRPM8
siCTRL
siTRPM8

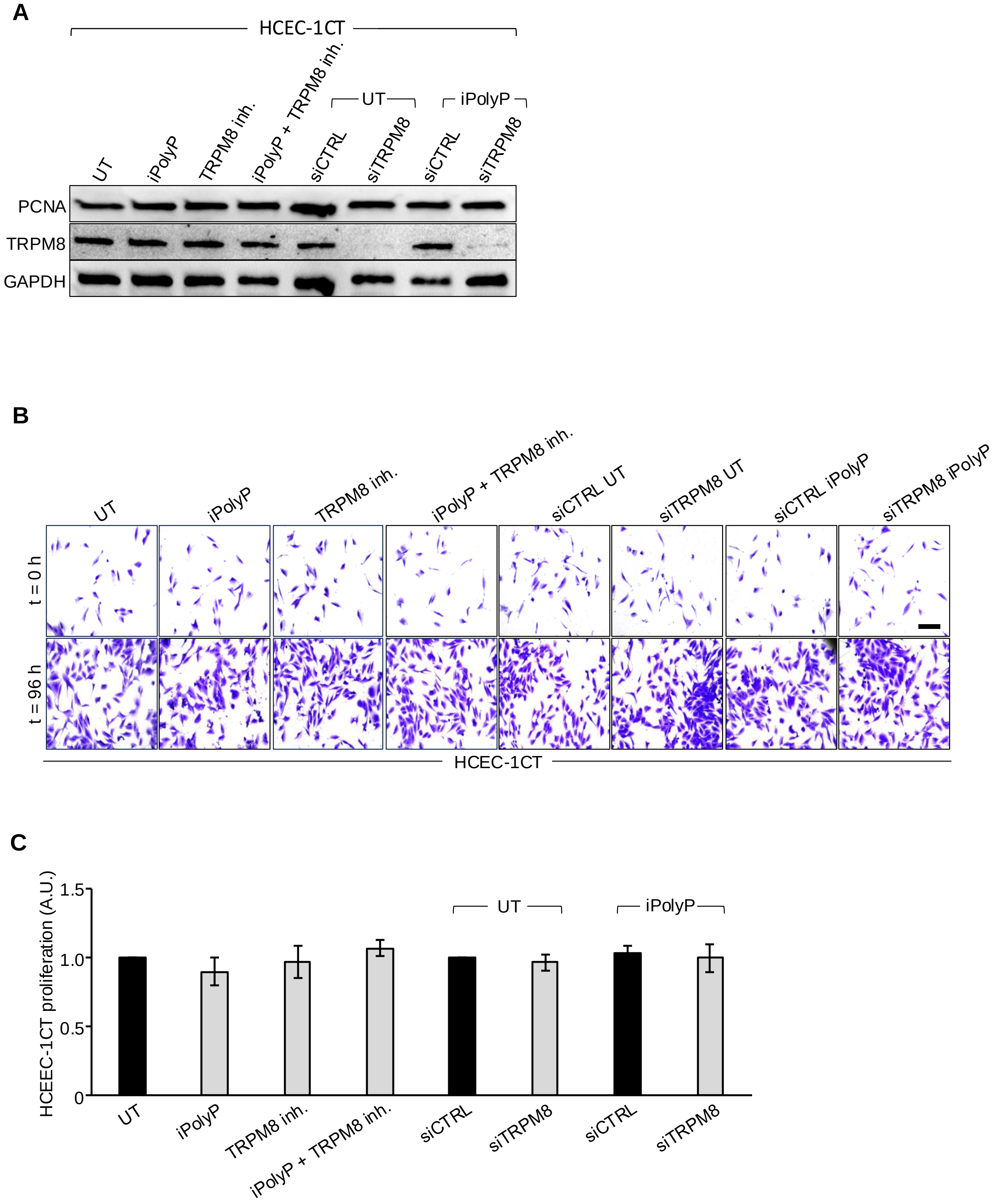
A
HCEC-1CT
UT
iPolyP
TRPM8 inh.
iPolyP + TRPM8 inh.
UT
iPolyP
siCTRL
siTRPM8
siCTRL
siTRPM8
PCNA
TRPM8
GAPDH
B
UT
iPolyP
TRPM8 inh.
iPolyP + TRPM8 inh.
siCTRL UT
siTRPM8 UT
siCTRL iPolyP
siTRPM8 iPolyP
t = 0 h
t = 96 h
HCEC-1CT
C
HCEEC-1CT proliferation (A.U.)
1.5
1.0
0.5
0
UT
iPolyP
UT
iPolyP
TRPM8 inh.
iPolyP + TRPM8 inh.
siCTRL
siTRPM8
siCTRL
siTRPM8

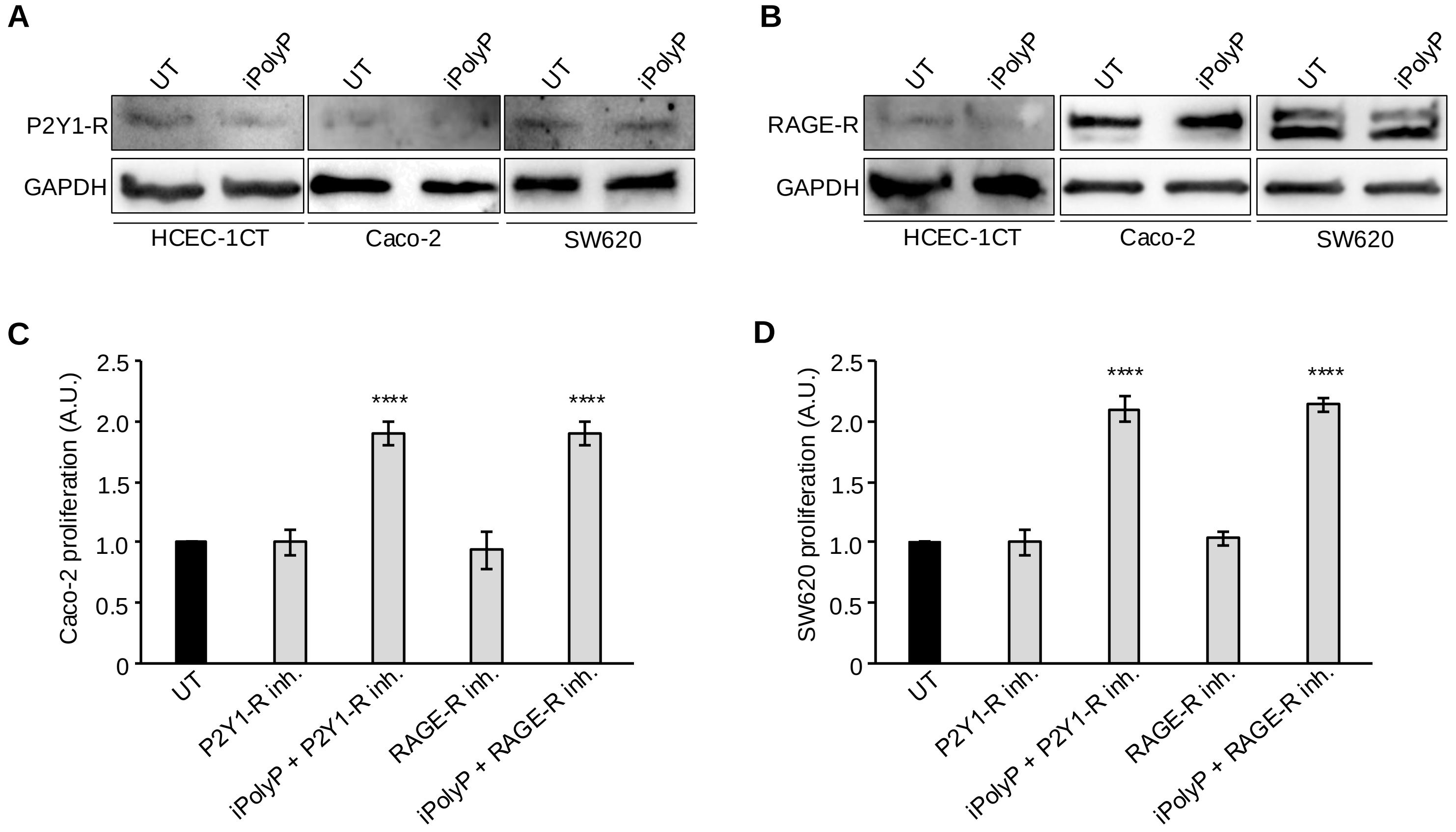
A
B
C
D
UT
iPolyP
P2Y1-R
GAPDH
RAGE-R
HCEC-1CT
Caco-2
SW620
Caco-2 proliferation (A.U.)
SW620 proliferation (A.U.)
2.5
2.0
1.5
1.0
0.5
0
****
UT
P2Y1-R inh.
iPolyP + P2Y1-R inh.
RAGE-R inh.
iPolyP + RAGE-R inh.

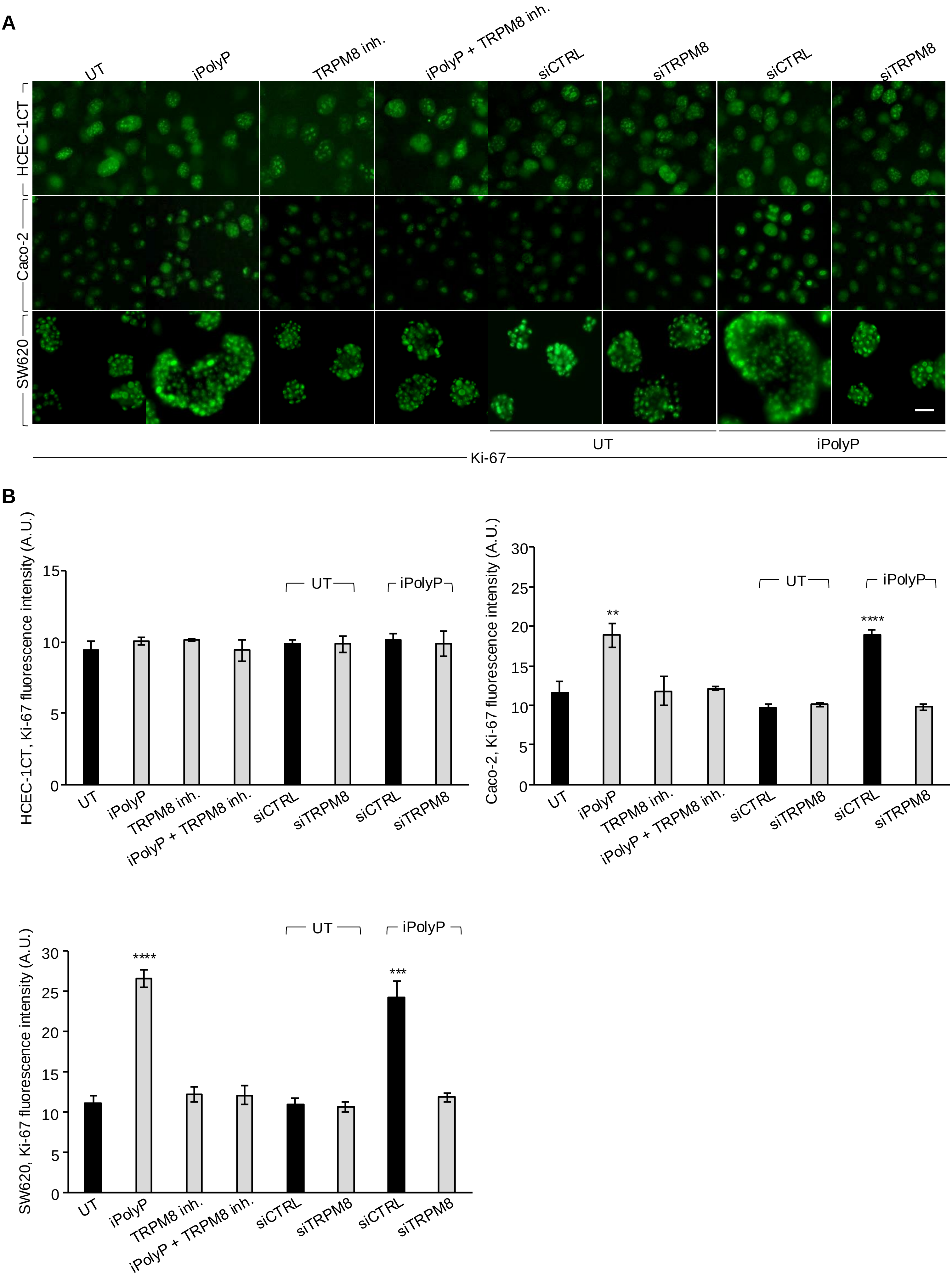
A
UT
iPolyP
TRPM8 inh.
iPolyP + TRPM8 inh.
siCTRL
siTRPM8
siCTRL
siTRPM8
HCEC-1CT
Caco-2
SW620
UT
iPolyP
Ki-67
B
HCEC-1CT, Ki-67 fluorescence intensity (A.U.)
15
10
5
0
UT
iPolyP
UT
iPolyP
TRPM8 inh.
iPolyP + TRPM8 inh.
siCTRL
siTRPM8
siCTRL
siTRPM8
Caco-2, Ki-67 fluorescence intensity (A.U.)
30
25
20
15
10
5
0
UT
iPolyP
**
****
UT
iPolyP
TRPM8 inh.
iPolyP + TRPM8 inh.
siCTRL
siTRPM8
siCTRL
siTRPM8
SW620, Ki-67 fluorescence intensity (A.U.)
30
25
20
15
10
5
0
UT
iPolyP
****
***
UT
iPolyP
TRPM8 inh.
iPolyP + TRPM8 inh.
siCTRL
siTRPM8
siCTRL
siTRPM8

A

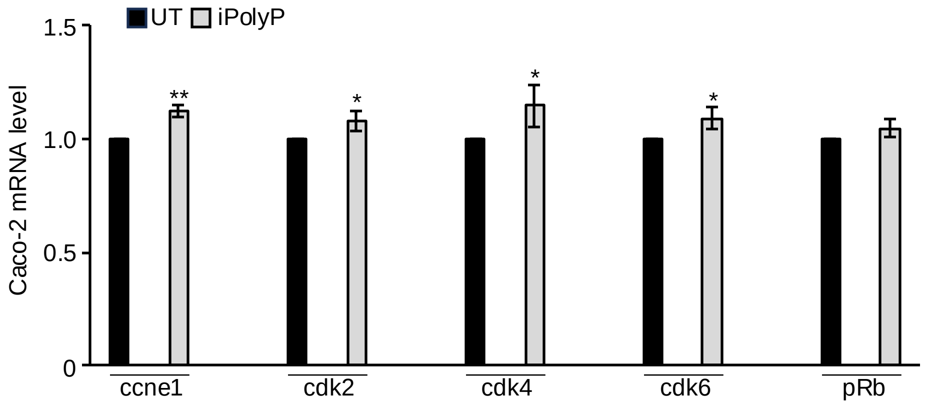

Supplementary Figure S1. **P2Y1 receptor and RAGE receptor level are not altered in CRC**. **A**. Cellular extracts from 4 human biopsies (Patients, Pt), where Tumoral sample (T) was plotted against the Peritumoral (P) counterpart of the same patient, were analyzed by immunoblotting for P2Y1 receptor (P2Y1-R) expression level. Actin was used as loading control for the normalization. **B**. Cellular extracts from 4 human biopsies, where Tumoral sample (T) was plotted against the Peritumoral (P) counterpart of the same patient, were analyzed by immunoblotting for RAGE receptor (RAGE-R) expression level. Actin was used as loading control for the normalization.

Supplementary Figure S2. **iPolyP enhances PCNA expression and promotes SW620 colorectal cancer proliferation**. **A**. Cellular extracts from WT and siRNA-mediated TRPM8 knockdown SW620 cell lines were analyzed by immunoblotting for PCNA expression level. GAPDH was used as loading control. **B**. Representative micrographs of the crystal violet assay performed on WT and siRNA-mediated TRPM8 knockdown SW620 cell lines upon treatment for 96 h with iPolyP, TRPM8 inhibitor or both. Scale bar 10 µm. Images are representative of three independent experiments. **C**. Statistical analysis of the crystal violet assay by Student's t-test, respectively for panel **B**, (*** $p < 0.001$ and **** $p < 0.0001$). Fold changes versus control, untreated (UT), normalized to 1. Data are presented as mean ± SD for triplicate wells from three independent experiments.

Supplementary Figure S3. **iPolyP does not compromise HCEC-1CT cell proliferation**. **A**. Cellular extracts from WT and siRNA-mediated TRPM8 knockdown HCEC-1CT cell lines were analyzed by immunoblotting for PCNA expression level. GAPDH was used as loading control. **B**. Representative micrographs of the crystal violet assay performed on WT and siRNA-mediated TRPM8 knockdown HCEC-1CT cell lines upon treatment for 96 h with iPolyP, TRPM8 inhibitor or both. Scale bar 10 µm. Images are representative of three independent experiments. **C**. Statistical analysis of the crystal violet assay by Student's t-test, respectively for panel **B**. Fold changes versus control, untreated (UT), normalized to 1. Data are presented as mean ± SD for triplicate wells from three independent experiments.

Supplementary Figure S4. **iPolyP does not alter the expression level of RAGE-R or P2Y1-R, not involved in iPolyP-mediated colorectal cancer cellular proliferation**. **A**. Cellular extract from HCEC-1CT, Caco-2 and SW620 cell line were analyzed by immunoblotting for P2Y1-R expression level, with and without iPolyP treatment. GAPDH was used as loading control. **B**. Cellular extract from HCEC-1CT, Caco-2 and SW620 cell line were analyzed by immunoblotting for RAGE-R expression level, with and without iPolyP treatment. GAPDH was used as loading control. **C**. Statistical analysis of the crystal violet assay by Student's t-test, performed with RAGE-R and P2Y1-R inhibitors on Caco-2 cell line and **D**. Statistical analysis of the crystal violet assay by Student's t-test, performed with RAGE-R and P2Y1-R inhibitors on SW620 cell line, (**** $p < 0.0001$); untreated, UT, normalized to 1. Data are presented as mean ± SD for triplicate wells from three independent experiments.

Supplementary Figure S5. **iPolyP induces nuclear protein Ki-67 expression via TRPM8 in Caco-2 and SW620 cell line**. **A**. Representative immunofluorescence images on WT HCEC-1CT (upper panel), Caco-2 (middle panel) and SW620 (lower panel) cell line and their corresponding siRNA-mediated TRPM8 knockdown, showing the expression of the proliferation marker Ki-67 upon treatment with iPolyP, TRPM8 inhibitor or iPolyP + TRPM8 inhibitor for 72 h. Scale bar 10 µm. Images are representative of three independent experiments. **B**. Fold changes versus control, untreated (UT). Statistical analysis performed by Student's t-test, relative to panel A (** $p < 0.01$, *** $p < 0.001$, **** $p < 0.0001$). Data are presented as mean ± SD for triplicate wells from three independent experiments.

Supplementary Figure S6. iPolyP enhances the expression level of Cyclin E and Cyclin-dependent kinase genes implicated in different phases of the cell cycle. **A**. Real-Time PCR on iPolyP-treated Caco-2 cell line for 72 h on cyclins implicated in difference phases of the cell cycle; untreated, UT, samples were normalized to 1. Statistical analysis was performed by Student's *t*-test (* $p < 0.05$, ** $p < 0.01$).
